# Isoquercitrin induces apoptosis and autophagy in hepatocellular carcinoma cells via AMPK/mTOR/p70S6K signaling pathway

**DOI:** 10.18632/aging.202237

**Published:** 2020-11-29

**Authors:** Liyan Shui, Weina Wang, Mingjie Xie, Bingjue Ye, Xian Li, Yanning Liu, Min Zheng

**Affiliations:** 1 State Key Laboratory for Diagnosis and Treatment of Infectious Diseases, National Clinical Research Center for Infectious Diseases, Collaborative Innovation Center for Diagnosis and Treatment of Infectious Diseases, The First Affiliated Hospital, College of Medicine, Zhejiang University, Hangzhou 310003, China; 2 Zhejiang Provincial Key Laboratory of Horticultural Plant Integrative Biology, Zhejiang University, Hangzhou 310058, China

**Keywords:** isoquercitrin, autophagy, hepatocellular carcinoma, apoptosis, AMPK

## Abstract

Hepatocellular carcinoma (HCC) is an aggressive malignancy with high rates of metastasis and relapse. Isoquercitrin (ISO), a natural flavonoid present in the Chinese bayberry and other plant species, reportedly exerts notable inhibitory effects on tumor cell proliferation, though the mechanism is unknown. In the present study, we exposed HepG2 and Huh7 human liver cancer cells to ISO and examined the roles of autophagy and apoptosis in ISO-mediated cell death. We found that ISO exposure inhibited cell viability and colony growth, activated apoptotic pathway, and triggered dysregulated autophagy by activating the AMPK/mTOR/p70S6K pathway. Autophagy inhibition using 3-methyladenine (3-MA) or Atg5-targeted siRNA decreased the Bax/Bcl-2 ratio, caspase-3 activation, and PARP cleavage and protected cells against ISO-induced apoptosis. Moreover, autophagy inhibition reversed the upregulation of AMPK phosphorylation and downregulation of mTOR and p70S6K phosphorylation elicited by ISO. By contrast, application of a broad-spectrum caspase inhibitor failed to inhibit autophagy in ISO-treated cells. These data indicate that ISO simultaneously induced apoptosis and autophagy, and abnormal induction of autophagic flux contributed to ISO-triggered caspase-3-dependent apoptosis.

## INTRODUCTION

Hepatocellular carcinoma (HCC), the most common primary liver malignancy, is the second leading cause of cancer-related death in males and the sixth in females [1]. Most HCC patients are diagnosed at an advanced stage, where invasion and metastasis greatly limit treatment options [2]. Even early HCC cases that received surgical resection, chemotherapy, or liver transplantation are still subject to numerous side effects and frequent relapse, rendering HCC one of the most lethal cancers. Given the poor clinical outcomes, it is critical to identify novel effective therapeutic strategies for HCC.

Most chemotherapy agents exploit apoptosis, a programmed cell death process, to hinder the development and progression of cancer [3]. An intricate relationship exists between apoptosis and autophagy, an essential catabolic process that serves to preserve cell viability under cellular stress [4]. Autophagy can be activated in many physiological and pathological contexts and is defined by the degradation and recycling of unfolded and aged proteins, as well as organelles, to maintain cellular homeostasis and prevent cellular damage against various stressors, including metabolic, hypoxic, chemotherapeutic, and detachment-induced stress [5]. Accordingly, autophagy has emerged as a key mechanism promoting tumor cell survival and resistance to cancer therapies. However, recent studies have shown that compared with normal cells, autophagy is generally reduced in many cancer cells [6], and suggested the relevance of various anticancer therapies that stimulate autophagy as a mean to induce tumor cell death [7–11]. The crosstalk between autophagy and apoptosis, essential for the maintenance of cellular homeostasis [12], is regulated by an extensive molecular interplay influenced by cell type, tissue microenvironment, and the specific conditions encountered by cells [13, 14]. Thus, research has shown that autophagy can inhibit apoptosis and promote cell survival, or may under certain conditions stimulate apoptosis and even trigger a distinct cell death program known as autophagy-related cell death [13].

The Chinese bayberry (Myrica rubra Sieb. et Zucc.) is a subtropical fruit tree, widely distributed in the hilly regions of Southern China, with a long cultivation history and multiple uses in Traditional Chinese medicine [15, 16]. Our previous study has identified isoquercitrin (ISO; quercetin-3-O-glucoside) as a main flavonoid of Chinese bayberry extracts and showed that it mediates significant enhancement of glucose consumption in HepG2 cells [17]. Other pharmacological studies have demonstrated that the M. rubra extract has powerful inhibitory actions on oxidative stress [18], excessive inflammatory responses [19], and tumor cell proliferation [20]. However, whether ISO can prevent tumor growth by stimulating autophagy remains unclear. Therefore, the present study aimed to explore the potential involvement of autophagy and its relation with apoptosis triggered by ISO on HCC cells.

## RESULTS

### Isoquercitrin inhibits HCC cell growth

**Figure 1:**
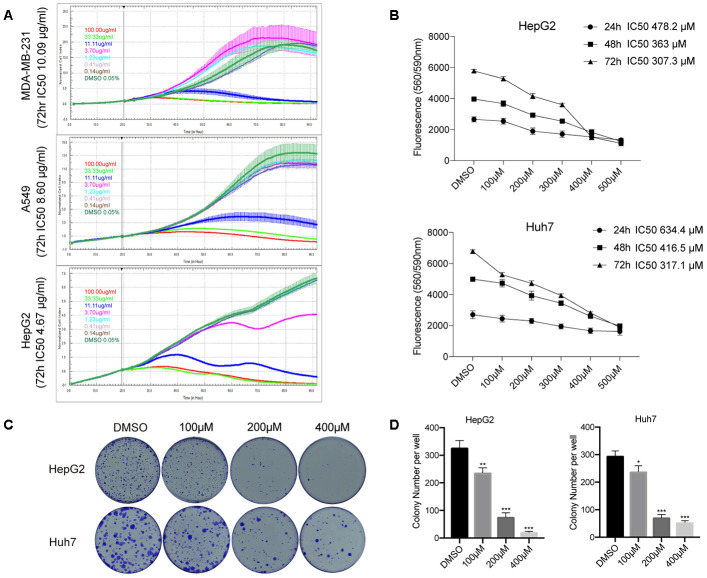
**ISO inhibits HCC cell growth.** (**A**) RTCA of proliferation of HCC cells treated with different concentration of CCE. (**B**) CellTiter-Blue viability assay results from HepG2 and Huh7 cells treated with 100-500 μM ISO or 0.2% dimethyl sulfoxide (DMSO; vehicle) for 24, 48, and 72 h. (**C**) Representative images of HepG2 and Huh7 cell colonies. Cells were treated with ISO and allowed to grow for 2 weeks before quantification of cell colonies stained with crystal violet. (**D**) Quantification of crystal violet-stained cell colonies. Values represent mean ± SD; *p < 0.05, **p < 0.01, ***p < 0.001.

The Chinese bayberry fruit has a diverse flavonoid composition responsible for its various medicinal activities. To investigate the potential anti-cancer effects of Chinese bayberry crude extracts (CCE), we treated several human cancer cell lines (MDA-MB-231, A549, and HepG2) with various concentrations of CCE (0.14, 0.41, 1.23, 3.70, 11.11, 33.33, and 100.00 μg/mL) for 72 h and assessed cell viability through the RTCA assay. As shown in Figure 1A, CCE reduced cell viability dose-dependently (MDA-MB-231, IC50 = 10.09 μg/mL; A549, IC50 = 8.6 μg/mL; HepG2, IC50 = 4.67 μg/mL), whereas no significant effect on cell growth was observed in control cultures treated with drug vehicle (DMSO 0.05%).

Using LC-ESI-Q-TOF-MS and HPLC, our previous study identified quercetin-3-O-glucoside [Q3G, isoquercitrin (ISO)] as one of the main flavonoids contributing to CCE’s antiproliferative activity on tumor cells [17]. Therefore, the potential therapeutic value of ISO against liver cancer was examined in subsequent experiments. CellTiter-Blue assays were performed to determine the anti-proliferative effect of ISO on HCC cells (HepG2 and Huh7 cell lines). The results indicated that ISO exposure suppressed the proliferation of HepG2 cells (IC50 = 307.3-478.2 μM) and Huh7 cells (IC50 = 317.1- 634.4 μM) after 24–72 h treatment (Figure 1B). Furthermore, we assessed ISO’s long-term growth inhibitory effect through colony formation assays. After 14 days of culture, colony formation was significantly inhibited in ISO-treated cells (Figure 1C, 1D). These data indicate that ISO inhibits cell growth and colony formation in HCC cells.

### Isoquercitrin induces apoptosis in HCC cells

**Figure 2:**
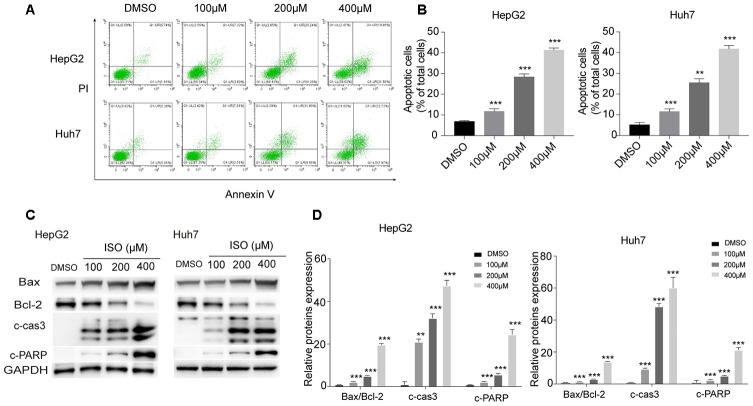
**ISO induces apoptosis in HepG2 and Huh7 cells.** (**A**) Apoptosis detection by Annexin V/PI staining and flow cytometry in HepG2 and Huh7 cells treated with ISO for 48 h. Criteria were set to distinguish between viable (bottom left), early apoptotic (bottom right), late apoptotic (top right), and necrotic (top left) cells. (**B**) Statistical analysis of apoptosis rate. (**C**) Western blotting analysis of apoptosis-related proteins. GAPDH was used as loading control. (**D**) Densitometric analysis of the data shown in (**C**). Values represent mean ± SD; *p < 0.05, **p < 0.01, ***p < 0.001.

Next, we examined by Annexin V-FITC/PI staining whether ISO exposure triggers apoptosis in HCC cells. As shown in Figure 2A, 2B, a significant increase in both early and late apoptosis rates was observed in ISO-treated HepG2 and Huh7 cells. To confirm this effect, we analyzed the expression of apoptosis-related proteins by western blotting. As shown in Figure 2C, 2D, ISO treatment (48 h) led to dose-dependent accumulation of both active (cleaved) caspase-3 and cleaved PARP and increased the Bax/Bcl-2 ratio. These results reveal that ISO inhibits proliferation and triggers apoptosis of HCC cells in a dose-dependent way.

### Isoquercitrin induces autophagy in HCC cells

**Figure 3:**
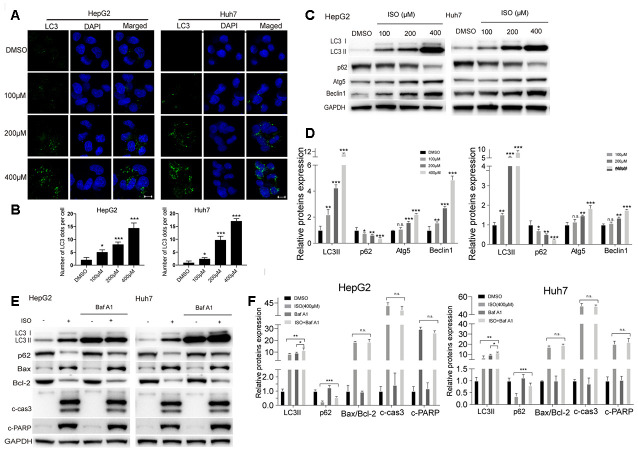
**ISO induces autophagy in HCC cells.** (**A**) Immunofluorescent detection of LC3 in HepG2 and Huh7 cells treated with ISO for 48 h. Nuclei were stained with DAPI. Scale bar: 100 μm. (**B**) The number of LC3 dots per cell were quantified using ImageJ. (**C**) Western blotting detection of autophagy-related proteins in HepG2 and Huh7 cells treated with ISO for 48 h. (**D**) Densitometric analysis of the data shown in (**C**). (**E**, **F**) Western blot analysis of LC3, p62, Bax, Bcl-2, cleaved caspase-3, and cleaved PARP expression in HepG2 and Huh7 cells treated with 400 μM ISO for 48, alone or after 2-h pre-treatment with 100 nM Bafilomycin A1. GAPDH was used as loading control. Values represent mean ± SD; *p < 0.05, **p < 0.01, ***p < 0.001.

Our preliminary experiments showed that an increase of GFP-LC3 puncta, denoting formation of autophagic vacuoles, occurred in HeLa cells treated with ISO (Supplementary Figure 1). Based on this observation, we analyzed LC3 puncta density and LC3-II levels, two variables positively correlated with autophagosome formation, in HCC cells exposed to ISO. Using immunofluorescence, a characteristic punctate LC3 pattern was detected in ISO-treated cells (Figure 3A, 3B). Meanwhile, western blot analysis of autophagy-related makers indicated a dose-dependent increase in LC3, Atg5, and Beclin-1, and a decrease in p62/sequestosome-1 (p62) expression, in HepG2 and Huh7 cells treated with ISO for 48 h (Figure 3C, 3D).

Autophagic flux comprises a sequence of events initiated by autophagosome formation and leading to degradation of autophagic substrates [21]. Accumulation of autophagosomes and increased levels of LC3-II indicate either activation of autophagic induction or blockade of downstream autophagic degradation steps [22, 23]. To determine whether ISO-mediated accumulation of LC3-II and increased LC3 puncta are due to the activation of autophagy or blockade of lysosomal degradation, we monitored autophagic flux after co-treatment with Bafilomycin A1 (Baf A1) which blocks autophagosome-lysosome fusion. Immunoblot analyses indicated that LC3-II levels were higher in HCC cells co-treated with ISO and Baf A1 than in those treated with ISO alone (Figure 3E, 3F). In turn, the expression of p62/sequestosome-1 (p62), a well-known autophagic cargo protein [24], was decreased following ISO treatment and restored by co-exposure to Baf A1 (Figure 3E, 3F). This evidence indicated that p62 is rapidly degraded during ISO-induced autophagy. Furthermore, p62 levels were restored to a lesser extent in Baf A1 co-treated cells than in cells treated with Baf A1 alone. Meanwhile, no obvious difference in activated caspase-3 levels was detected between cells treated with ISO alone and those exposed also to Baf A1 (Figure 3E, 3F). Together, these results strongly indicate that ISO induces autophagy in both HepG2 and Huh7 cells.

### Isoquercitrin-induced autophagy is mediated by AMPK/mTOR/p70S6K signaling

**Figure 4:**
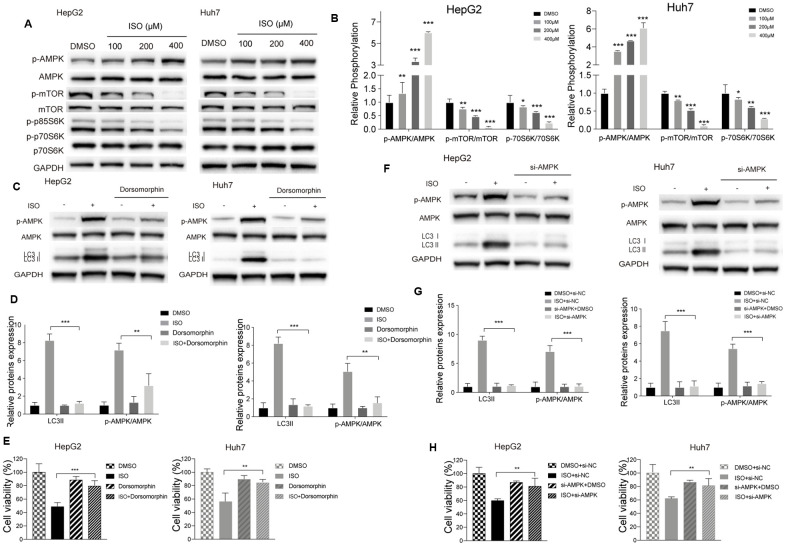
**ISO induces autophagy through the AMPK/mTOR/p70S6K signaling pathway.** (**A**) Western blotting analysis of AMPK, mTOR, p70S6K and their phosphorylated forms in HepG2 and Huh7 cells treated with increasing concentrations of ISO or 0.2% DMSO (vehicle) for 48 h. (**B**) Densitometric analysis of autophagy-related proteins. p-AMPK, AMPK, and LC3-II expression was detected after blocking AMPK activation with dorsomorphin (**C**, **D**) or si-AMPK (**F**, **G**); GAPDH served as loading control. (**E**, **H**) CellTiter-Blue viability assay results from HCC cells treated with 400 μM ISO for 48 h in the presence or absence of dorsomorphin or si-AMPK. Values represent mean ± SD; *p < 0.05, **p < 0.01, ***p < 0.001.

Since activation of AMP-dependent protein kinase (AMPK) and inhibition of mammalian target of rapamycin (mTOR) is a main mechanism of autophagy activation [25–28], we examined whether changes in the activation status of the AMPK/mTOR/p70S6 signaling cascade occurred during ISO-induced autophagy. As expected, ISO treatment led to dose-dependent activation of AMPK and decreased the phosphorylation of mTOR and its downstream substrate p70/p85S6 kinase (p70/p85S6K) (Figure 4A), without affecting total AMPK and mTOR levels (Figure 4B). Next, we tested whether AMPK inhibition affected the increase in LC3-II elicited by ISO in both HCC cell lines. Western blot assays showed that pharmacological blockade of AMPK activation with dorsomorphin markedly inhibited AMPK phosphorylation and significantly attenuated LC3-II expression in ISO-treated cells (Figure 4C, 4D). To confirm that ISO exposure induces accumulation of LC3B-II through activation of AMPK signaling, we performed siRNA-mediated AMPK knockdown, and confirmed through western blotting that this maneuver also inhibited the increase in LC3B-II (Figure 4F, 4G). Importantly, both dorsomorphin treatment and AMPK knockdown significantly reversed ISO-induced cytotoxicity (Figure 4E, 4H). These findings support our hypothesis that ISO-induced autophagy proceeds via AMPK/mTOR/p70S6K signaling and suggests that autophagy is a key determinant of ISO-mediated cell death.

### Autophagy activation underlies ISO-mediated HCC cell death

**Figure 5:**
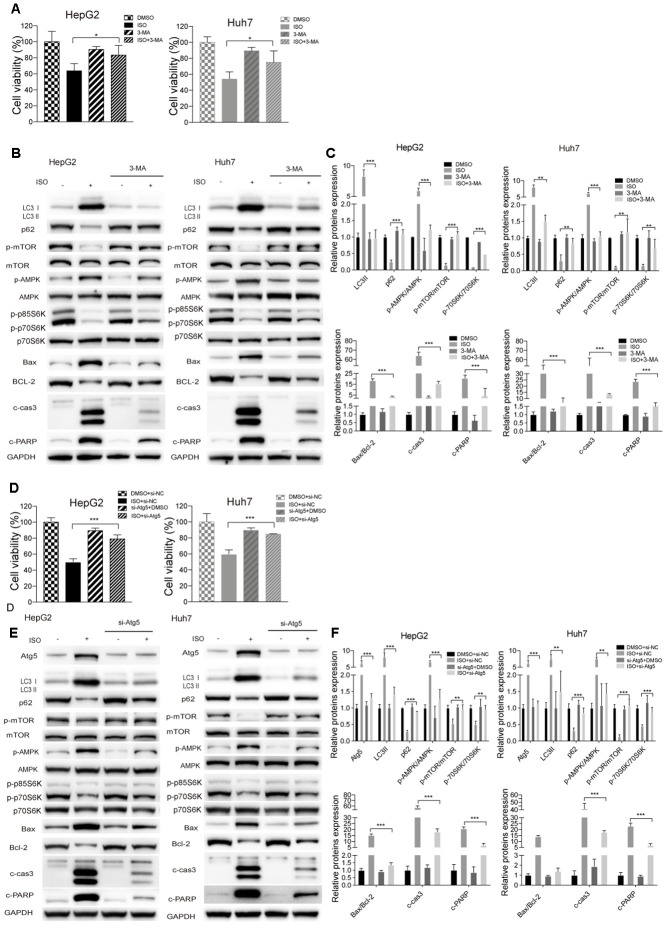
**Autophagy inhibition attenuates ISO-induced cell death.** HepG2 and Huh7cells were pre-treated with 3-MA (5 mM) or vehicle for 2 h or transfected with si-Atg5 or si-NC for 6 h, and then treated with ISO (400 μM) for 48 h. (**A**, **D**) CellTiter-Blue viability assay results. Data are expressed as percentage relative to untreated control cells. (**B**, **E**) Western blotting analysis of LC3, p62, p-mTOR, mTOR, p-AMPK, AMPK, p-p70S6K, p70S6K, Bax, Bcl-2, cleaved caspase-3, and cleaved PARP expression. (**C**, **F**) Densitometric analysis of protein expression data. Data are expressed as fold change relative to values from untreated control cells after normalization against GAPDH. Values are expressed as mean ± SD; *p < 0.05; **p < 0.01; ***p < 0.001.

Autophagy may be either a cytoprotective or cytotoxic phenomenon, depending on the cellular context and the nature of the stress endured by the cells [29–31]. To confirm the role of autophagy in ISO-induced cell death, pharmacological (3-MA) and genetic (si-Atg5) inhibition of autophagy was performed in HCC cells. CellTiter-Blue assays showed that both maneuvers increased resistance against ISO-induced toxicity in both HepG2 and Huh7 cells (Figure 5A, 5D). To further determine the correlation between ISO-mediated autophagic induction and apoptotic cell death, we analyzed LC3B-II processing, PARP cleavage, and caspase-3 levels by western blot. Compared to ISO-treated control cells, a decrease in LC3B-II expression paralleled by an increase in p62 levels was observed following ISO exposure in HepG2 and Huh7 cells pre-treated with 3-MA or si-Atg5 (Figure 5B, 5E). These cells showed also a marked reduction in cleaved PARP, cleaved-caspase-3, and the Bax/Bcl-2 ratio, indicating that autophagy inhibition led to apoptosis blockade (Figure 5B, 5E). In addition, the changes induced by ISO in AMPK/mTOR/p70S6 phosphorylation status were prevented by co-treatment with 3-MA or si-Atg5 (Figure 5B, 5E). Quantitative results for these western blot assays are shown in Figure 5C, 5F.

### ISO-triggered autophagy precedes caspase-dependent apoptosis

**Figure 6:**
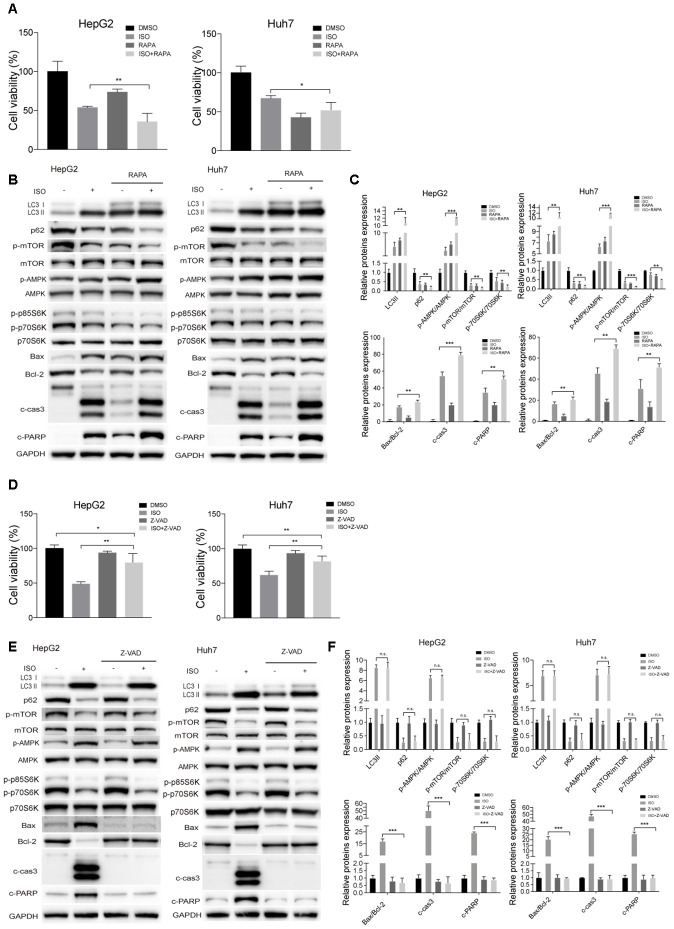
**ISO-triggered autophagy in HCC cells is promoted by rapamycin and not prevented by Z-VAD-FMK.** HepG2 and Huh7cells were treated with 400 μM ISO for 48 h in the presence or absence of 100 nM rapamycin (RAPA) or 100 μM z-VAD-FMK (zVAD). (**A**, **D**) CellTiter-Blue viability assay results. (**B**, **E**) Immunoblot analyses of LC3, p62, p-mTOR, mTOR, p-AMPK, AMPK, p-p70S6K, p70S6K, Bax, Bcl-2, cleaved caspase-3, and cleaved PARP expression. (**C**, **F**) Densitometric analysis of protein expression data. Data are expressed as fold change relative to values from untreated control cells after normalization against GAPDH. Values are expressed as mean ± SD; *P < 0.05; **P < 0.01; ***p < 0.001.

To further validate the correlation between ISO-triggered autophagy and apoptosis induction, the mTOR inhibitor rapamycin (RAPA) was used to stimulate autophagy in HCC cells. RAPA addition decreased cell viability and strengthened the inhibitory effect of ISO on cell growth (Figure 6A) while markedly increasing, either alone or with ISO, cleaved caspase-3 and cleaved PARP levels, as well as the Bax/Bcl-2 ratio. Furthermore, RAPA co-incubation potentiated LC3II expression and AMPK phosphorylation, and further suppressed p62 expression and both mTOR and p70S6K phosphorylation (Figure 6B, 6C). These data reveal that ISO and rapamycin exert a synergistic effect on apoptosis induction, and that suppression of autophagy diminishes ISO-induced apoptosis in HepG2 and Huh7 cells.

We then used the pan-caspase inhibitor Z-VAD-FMK to examine whether caspase activation would affect ISO-induced autophagy. As shown in Figure 6D, the inhibition of cell viability caused by ISO was decreased in the presence of Z-VAD-FMK, while Z-VAD-FMK alone did not have any cytotoxic effects. In turn, in ISO-treated cells, apoptotic hallmarks, including the Bax/Bcl-2 ratio, cleaved caspase-3, and PARP cleavage, were also suppressed by ZVAD-FMK (Figure 6E, 6F). However, Z-VAD-FMK had little effect on LC3-II and p62 levels. These data strongly indicate that autophagic events precede ISO-induced apoptosis in HCC cells.

## DISCUSSION

**Figure 7:**
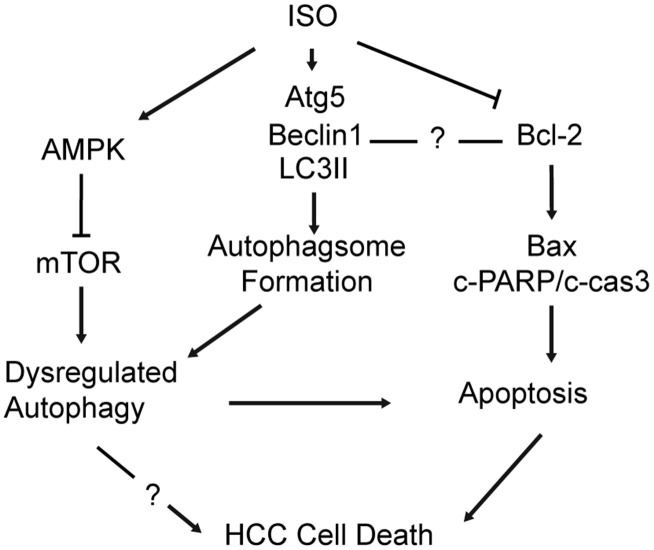
**Schematic diagram of the mechanism underlying ISO’s anti-HCC effects.** ISO exposure activates AMPK and inhibits mTOR signaling in HCC cells, leading to upregulation of Atg5, Beclin-1 and LC3B-II and autophagosome formation (1). ISO-induced cell death is effectively alleviated by autophagy blockade (3-MA; si-Atg5) and further enhanced by autophagy stimulation (RAPA), indicating that autophagy activation contributes to ISO-induced cell death. In parallel, activation of the mitochondrial-dependent intrinsic apoptotic pathway is evidenced by a concomitant increase in cleaved caspase-3, cleaved PARP, and the Bax/Bcl-2 ratio (2).

Clinical outcomes of HCC remain dismal, since approximately only one-third of patients are eligible for curative therapies such as surgical resection, percutaneous ablation, or liver transplantation [32]. In the present study we investigated the potential of ISO as a therapeutic agent in liver cancer. Our data revealed that ISO exerted growth inhibitory effects on HCC cells by coordinating the crosstalk between autophagy and apoptosis (Figure 7). At the molecular level, ISO actions were characterized by up-regulation of active caspase-3 and cleaved PARP and increased Bax/Bcl-2 ratio, indicative of apoptosis, concomitant with enhanced LC3-II expression and p62 degradation, reflecting induction of autophagy. Since autophagy inhibition attenuated apoptosis in ISO-treated HepG2 and Huh7 cells, we conclude that ISO-induced autophagy exerted a cytotoxic rather than a cytoprotective effect.

Phytonutrients are receiving increasing attention as potential anti-tumor agents. ISO, a flavonoid found in several plant species, has shown to suppress cancer cell proliferation and migration by targeting MAPK signaling in liver cancer [33] and Wnt/β-catenin signaling in colon cancer [34]. Most notably, ISO has been demonstrated to induce apoptosis in liver cancer cells [33]. The fact that apoptosis often occurs simultaneously with autophagy prompted us to explore whether autophagy is also involved in ISO’s anti-HCC effect. Autophagy is a catabolic process in which organelles and macromolecules are degraded and recycled to sustain metabolism [4]. However, the exact role of autophagy in cancer is controversial, as both a protective effect against the cytotoxic effects of anti-cancer drugs, and promotion of cell death via apoptosis or distinct autophagy-related cell death pathways, have been observed in different tumor cells [13, 35]. Therefore, assessing the role of autophagy in a context-dependent manner is crucial to determine whether autophagy-targeted interventions can be exploited for anticancer therapy.

The complex molecular connections between autophagy and apoptosis, two evolutionarily conserved processes that are triggered in response to various stresses, remain incompletely understood [35]. Apoptosis and autophagy can cooperate, antagonize, or assist each other to differentially influence cell fate. These events may be concurrently triggered by common upstream signals; in other instances, they may represent instead mutually exclusive cellular responses [36]. For instance, it has been reported that vitamin K2 induces autophagy and apoptosis simultaneously in leukemia cells [37]. In turn, the survivin inhibitor YM155 inhibits the growth of PC3 and LNCaP prostate cancer cells by inducing autophagy-dependent apoptosis [38]. In our study, because HCC cell death depended on ISO-induced caspase-3 activation, it is unclear whether dysregulated autophagy itself directly led to autophagic cell death (ACD). Nevertheless, we explicitly showed that dysregulated autophagy contributed to ISO- triggered caspase-3-dependent apoptosis. We confirmed this temporal cell death sequence by using pharmacological inhibition and genetic deletion experiments. For example, we demonstrated that cotreatment with 3-MA significantly blocked ISO-triggered caspase-3 activation and partially protected cells against ISO toxicity in HCC cells. We also found that ISO-induced caspase-3 activation, PARP cleavage, and Bax/Bcl-2 ratio were attenuated when the induction of autophagy was prevented by the genetic depletion of Atg5. However, incubation with the pan-caspase inhibitor Z-VAD-FMK did not completely block ISO-mediated HCC cell death, and had little effect on LC3-II levels. Therefore, our results show a strong causal relationship between autophagy and apoptotic cell death in which ISO-triggered dysregulated autophagy promotes caspase-dependent apoptotic cell death.

The AMPK/mTOR/p70S6K signaling pathway is a central regulator of autophagic flux [39, 40]. Upon metabolic stress, AMPK becomes activated and may trigger autophagy by phosphorylating and inactivating mTOR [41, 42], a core nutrient-sensing signaling complex whose activity regulates the balance between cell growth and cell death in response to different stimuli. Inhibition of mTOR kinase activity can promote autophagy by inhibiting its downstream substrate p70S6K [43, 44], restraining in turn its downstream effector protein Beclin-1, an essential autophagy regulator [45]. Therefore, we investigated the involvement of the AMPK/mTOR/p70S6K pathway on ISO-induced autophagy and apoptosis. Blocking the expression of AMPK, either pharmacologically or via siRNA, prevented ISO-induced HCC cell death. To better discriminate the role of mTOR in ISO- triggered autophagy, HCC cells were co-treated with the mTOR inhibitor RAPA, which led to a greater increase in ISO-induced LC3B-II levels in both cell lines. Furthermore, early-stage autophagy inhibitor 3-MA, mainly block the formation of autophagosomes through the inhibition of class III PtdIns 3-kinases, co-incubation attenuated LC3II expression and AMPK phosphorylation, and further rescued p62 expression and both mTOR and p70S6K phosphorylation. Because pharmacological effects might be insufficiently specific, to strengthen out hypothesis, the Atg5 siRNA, in which the early stage of autophagy was suppressed, were used. Consistently, the changes induced by ISO in AMPK/mTOR/p70S6 phosphorylation status were prevented by co-treatment with si-Atg5. These findings further indicated that the AMPK/mTOR/p70S6K pathway is involved in the activation of autophagy and apoptosis in HCC cells.

Research has shown that Bcl-2 and Beclin-1 mediate a close link between autophagy and apoptosis [46–48]. In a previous study, ISO inhibited the expression of Bcl-2 [49], suggesting that ISO is beneficial to inducing autophagy and apoptosis in HCC cells. Meanwhile, in Bcl-2-overexpressing breast cancer cells, inhibition of Bcl-2 by siRNA had no effect on apoptosis, but led instead to Beclin1 upregulation and autophagic tumor cell death [50]. Indeed, a complex interplay exists between Bcl-2 (and other proteins containing BH3 domains) and Beclin1, as part of a multilevel regulatory network linking autophagy and apoptosis through changes in protein expression levels, domain affinities, and phosphorylation events determining specific binding states [51]. Another key autophagic factor, Atg5, is also involved in the molecular network regulating autophagy and apoptosis [52, 53]. Atg5 can interact with FADD during IFN-gamma-induced autophagic cell death, eventually activating caspase-3 [54, 55]. Therefore, we examined the effect of Atg5 on apoptosis by detecting the expression of the apoptosis-related genes Bax, Bcl-2, and caspase-3. The Bax/Bcl-2 ratio decreased significantly in HCC cells following Atg5 knockdown, which indicates that autophagy inhibition via Atg5 silencing can prevent apoptosis, and further proved the slow growth of the Atg5 knockout cells was due to its own apoptosis and autophagy-induced apoptosis.

In conclusion, the present study revealed, for the first time, that ISO triggers HCC cell death by simultaneous induction of apoptosis and autophagy via AMPK activation and inhibition of mTOR/p70S6K signaling. These data shed light on the mechanisms underlying the antitumoral effects of ISO and suggest its potential therapeutic value as an inducer of both autophagy and apoptosis in HCC.

## MATERIALS AND METHODS

### Cell lines and cell culture

Human HCC cell lines HepG2 and Huh7 were obtained from the American Type Culture Collection (VA, USA). Cells were cultured at 37° C with 5% CO2 in Dulbecco’s Modified Eagle’s Medium (DMEM) (Gibco, CA, USA) with 10% fetal bovine serum (Gibco) and 1% penicillin/streptomycin (Invitrogen, MA, USA).

### Reagents and antibodies

Chinese bayberry crude extracts (CCE) were prepared as previously described [17]. Isoquercitrin and anti-LC3B antibody were purchased from Sigma-Aldrich (MO, USA). Antibodies against phospho-Ser2481-mTOR (Cat. 2974), mTOR (Cat. 2983), AMPKα (Cat. 5831), phospho-Thr172-AMPKα (Cat. 2535), cleaved-PARP (Cat. 5625), cleaved-caspase-3 (Cat. 9664), p62 (Cat. 8025), Beclin-1 (Cat. 3495), Atg5 (Cat. 2630), phospho-Thr389-p70S6 kinase (Cat. 9234), total p70S6 kinase (Cat. 9202), and GAPDH (Cat. 2118) were purchased from Cell Signaling Technology (MA, USA). Antibodies against Bax (Cat. ab32503) and Bcl-2 (Cat. ab182858) were obtained from Abcam (Cambridge, UK). 3-Methyladenine (3-MA), Bafilomycin A1 (Baf A1), Rapamycin (RAPA), z-VAD-FMK, and dorsomorphin were purchased from MCE (NJ, USA). Alexa Fluor 488 anti-rabbit IgG was bought from Invitrogen. Other chemicals and reagents were obtained from Sigma-Aldrich.

### Real-time cell analysis

Experiments using Real-Time Cell Analysis (RTCA) were performed at 37° C with 5% CO_2_ using an iCELLigence Analyzer (ACEA Biosciences Inc., CA, USA, Cat. 00380601120). Background measurements were conducted on E-plate L8 plates (ACEA Biosciences Inc., Cat. 00300600840) containing 200 μL DMEM. For cellular assays, 400 μL of a cell suspension (8,000 cells/well) containing various concentrations of CCE were added to plates and kept at room temperature (RT) for 30 min to allow cell deposition. Impedance values (cell index) were recorded every 15 min for 96 h. Every independent experiment was performed in triplicate. The slope of the cell index was calculated automatically by the RTCA software package 1.1.1 (ACEA, Biosciences Inc.).

### Cell viability assay

Cells were seeded at a density of 3.0 ×10^3^ cells/well in 96-well culture plates and allowed to attach overnight before being exposed to various concentrations of ISO for the indicated times. Cell viability was determined every day using a CellTiter-Blue assay kit (Promega, WI, USA) according to the instructions of the manufacturer.

### Colony formation assay

Cells were plated in 6-well plates at a density of 3,000 cells per well. ISO-containing media were refreshed every 3 days for 14 days. After this period, the cultures were washed with PBS once and the cell colonies stained with 0.1% crystal violet (Beyotime, Shanghai, China) for 10 min at RT. Images of each well were recorded with a digital camera and the number of colonies was counted with ImageJ software. Data represent the mean ± SD derived from at least 3 separate experiments.

### Apoptosis assay

Cell apoptosis was assessed in HCC cells treated with various concentrations of ISO for 48 h using a FITC Annexin V Apoptosis Detection Kit (BD Biosciences, CA, USA). according to the manufacturer’s instructions. Cells were analyzed on a FACScan flow cytometer (Beckman Coulter Inc., CA, USA) using CellQuest and FlowJo software.

### Western blotting

Western blotting was performed using a standard protocol. Briefly, HCC cells were harvested and lysed with 1% ice-cold SDS lysis buffer (Beyotime, Shanghai, China) include protease and phosphatase inhibitors (Roche), and protein concentration was quantified using a BCA Assay Kit (Thermo Fisher, DE, USA). Protein samples (30 μg) were subjected to 4-20% gradient PAGE gel (GenScript M42012) with Tris-MOPS running buffer, and transferred onto PVDF membranes (Millipore, MA, USA). The membranes were blocked with 5% non–fat milk at RT for 1 h and incubated sequentially with primary antibodies at 4° C overnight. Horseradish peroxidase-conjugated secondary antibodies (MultiSciences, Hangzhou, China) were added the following day for 1 h and then washed with TBST. Protein signals were detected using chemiluminescent ECL reagent (Thermo Scientific, USA), analyzed on an AlphaImager HP gel imaging system, and quantified with an image analysis software.

### Confocal immunofluorescence

For immunofluorescence staining, cells were cultured on cover slips, fixed with 4% paraformaldehyde for 15 min, and permeabilized with 0.1% Triton X-100. After blocking in 3% BSA for 30 min, primary anti-LC3B antibodies diluted in 1% BSA were added overnight at 4° C. After washing 3 times with PBS, slides were incubated with Alexa Fluor 488-conjugated secondary antibody (1:1000) for 1.5 h at RT. Cells were then counterstained with DAPI and LC3B puncta images were captured using an inverted confocal fluorescence microscope (Carl Zeiss, Germany).

### siRNA transfection

siRNAs targeting human Atg5 (si-Atg5), AMPK (si-AMPK), as well as scrambled nonspecific (control) siRNAs were obtained from RiboBio (Guangzhou, China). The sequences of the targeted siRNAs were: Atg5–1 GGATGCAATTGAAGCTCAT, Atg5–2 GGAAGCAGAACCATACTAT; AMPK-1 GCTTGATGCACACATGAAT, AMPK-2 GCTGCACCAGAAGTAATTT. Cells were seeded into 6-well plates and transiently transfected at approximately 60% confluence with 50 nM siRNA using Lipofectamine 3000 Transfection Reagent (Invitrogen) according to the manufacturer’s instructions. After a 6-h incubation in antibiotic-free medium the transfection medium was replaced by fresh medium, and 24 h later the cells were treated as indicated and then harvested for analysis.

### Statistical analysis

Statistical analyses were performed using GraphPad Prism software (GraphPad Software Inc., La Jolla, CA). Statistical comparisons were performed with one-way ANOVA followed by Dunnett’s t-test. P < 0.05 was defined as statistically significant. Data represent the mean ± SD derived from at least 3 separate experiments.

## Supplementary Material

Supplementary Figure 1
