# Supplementary figures and images for: Isoquercitrin induces apoptosis and autophagy in hepatocellular carcinoma cells via AMPK/mTOR/p70S6K signaling pathway
Source: Aging (Albany NY). 2020 Nov 29;12(23):24318–32. doi: 10.18632/aging.202237 (PMC7762471; doi:10.18632/aging.202237)

## SUPPLEMENTARY FIGURE

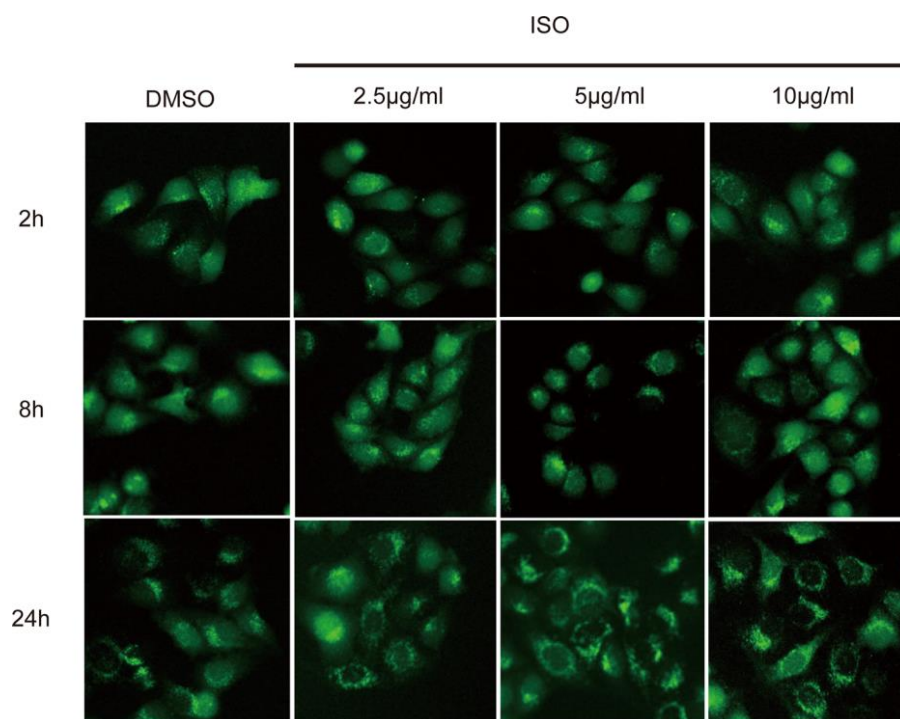

Supplementary Figure 1. GFP-LC3 puncta in ISO-treated Hela cells.

Supplement: Supplementary Figure 1 [file aging-12-202237-s001.pdf]
